# Neurogenic diabetes insipidus presenting in a patient with subacute liver failure: a case report

**DOI:** 10.1186/1752-1947-4-232

**Published:** 2010-07-30

**Authors:** Manu Shankar Hari, Anthony K Parsons, Andy K Burroughs, Steve Shaw, James O'Beirne, Banwari Agarwal

**Affiliations:** 1 Intensive Care Unit, The Royal Free Hospital NHS Trust, Pond Street, London, NW3 2QG, UK; 2 Liver Transplantation and Hepato-Biliary Medicine Unit, The Royal Free Hospital NHS Trust, Pond Street, London, NW3 2QG, UK

## Abstract

**Introduction:**

To the best of our knowledge, this is the first report in the literature of development of neurogenic diabetes insipidus in a patient with subacute liver failure.

**Case presentation:**

A 25-year-old man presented with subacute liver failure. While awaiting a liver transplant, the patient developed cerebral edema, which resulted in neurogenic diabetes insipidus secondary to cerebral edema. The patient died before the liver transplantation could be carried out.

**Conclusion:**

Neurogenic diabetes insipidus is well recognized in the neurosurgical population as a consequence of cerebral edema and increased intracranial pressure, both of which occur commonly in patients with subacute liver failure.

## Introduction

Cerebral edema occurs in patients presenting with fulminant liver failure, resulting in increased intracranial pressure (ICP). The incidence and severity of cerebral edema increases as the onset of liver failure becomes rapid. It occurs in up to 80% of patients with acute and hyperacute fulminant liver failure but less frequently (20%) in those with subacute fulminant liver failure. There is a significant association between the presence of cerebral edema and the development of central diabetes insipidus in patients with traumatic brain injury [1] and in postoperative neurosurgical patients. We present a case of neurogenic diabetes insipidus that developed during the course of subacute fulminant liver failure.

## Case presentation

A 25-year-old Nigerian man was admitted to our hospital's intensive care unit (ICU), after emergency tracheal intubation and ventilation for worsening encephalopathy and a deteriorating Glasgow Coma Scale score. The working diagnosis was seronegative hepatitis leading to subacute fulminant liver failure and grade three hepatic encephalopathy.

His admission to our hospital was preceded by an admission to a local hospital with a ten day history of painless jaundice and malaise, progressing to three days of nausea and vomiting. The patient had no history of encephalopathy or coagulopathy. Liver function tests on admission were abnormal (bilirubin, 381 μmol/L, alanine transferase [ALT], 684 U/L), but an ultrasound scan showed a normal liver. Autoimmune and viral screen results were negative except for anti-smooth muscle antibody, which was 1 in 40 dilutions. He was sickle cell negative, and virology screening results were also negative. He had coagulopathy with an International Normalized Ratio (INR) above ten, which responded to vitamin K and fresh-frozen plasma (FFP). The patient had no history of any recent foreign travel, intravenous (IV) drug use, excessive alcohol intake or any family history of liver or autoimmune disease. The patient was discharged home five days after admission with plans for outpatient follow-up.

Six weeks after the initial presentation, the patient was readmitted to the same local hospital with continuing malaise, reduced appetite, vomiting, abdominal pain, worsening jaundice and confusion. The liver function tests on this occasion were bilirubin, 597; ALT, 475; alkaline phosphatase (ALP), 859; albumin, nine; and INR above nine. Results of blood films for malaria were negative. The patient was intubated and ventilated in the ICU before being transferred to our hospital on three weeks after this admission. He was found to have grade three hepatic encephalopathy. A repeat ultrasound scan of his abdomen revealed mild hepatosplenomegaly. A computed tomography (CT) scan of his head before transfer was normal, and CT scans of his thorax and abdomen showed a normal-looking liver, splenomegaly and coeliac lymphadenopathy.

On admission to our ICU, the patient was hemodynamically stable and was ventilated on pressure-controlled synchronized intermittent mandatory ventilation (PCSIMV) to achieve normocapnia (PaCO_2_, 4.0-4.5 kPa). His urea and creatinine levels at admission were 2.8 mmol/L and 112 μmol/L, respectively. His INR was 4.4 after correction with FFP and vitamin K. Paracetamol levels were undetectable, and the serum lactate level was normal.

The patient was diagnosed with seronegative (virology screen negative) subacute (jaundice and encephalopathy > eight weeks) fulminant liver failure, such that he was listed for urgent liver transplantation as the satisfied the King's College criteria for transplantation in acute liver failure unrelated to paracetamol overdose [2].

After the patient was listed for urgent liver transplant, a retrograde jugular bulb catheter was inserted into the patient to monitor his jugular venous oxygen saturation and to optimize cerebral perfusion management. His jugular venous saturation was 91%, suggesting cerebral hyperemia. The management as per our ICU protocol included mild hypothermia (35°C), deep IV sedation and mean arterial pressure of below 85 mm Hg. This reduced his S_jv_O_2 _to 73%, which was followed by a sustained increase of more than 85%. The patient's serum ammonia level at this time was 170 μmol/L (reference range, 10-50 μmol/L). In keeping with our standard practice for the management of acute liver failure, an extradural Camino ICP bolt was inserted to monitor his ICP. The opening ICP was 15 mm Hg, which settled at 8 9 mm Hg with conservative management using a clear pressure waveform.

Over the next 24 hours, the patient became progressively polyuric, with a urine output ranging between 300 and 450 mL/h. He had not received mannitol or other diuretics during his admission to the ICU. His serum sodium level increased to 168 mmol/L, and his urea and glucose levels were at 3 mmol/L and 3.6 mol/L, respectively. His paired measured plasma was 368 mOsmol/L and urine osmolalities were 175 mOsmol/L. The polyuria was initially treated with water via nasogastric tube and with IV electrolyte-free solutions. A diagnosis of central diabetes insipidus was then made. Nephrogenic diabetes insipidus was excluded because of the absence of drugs known to cause it (the patient was being given piperacillin/tazobactam [Tazocin], fluconazole, pantoprazole and lactulose), normal blood levels of potassium and calcium ions, and the resolution of polyuria in response to desmopressin therapy.

**Figure 1:**
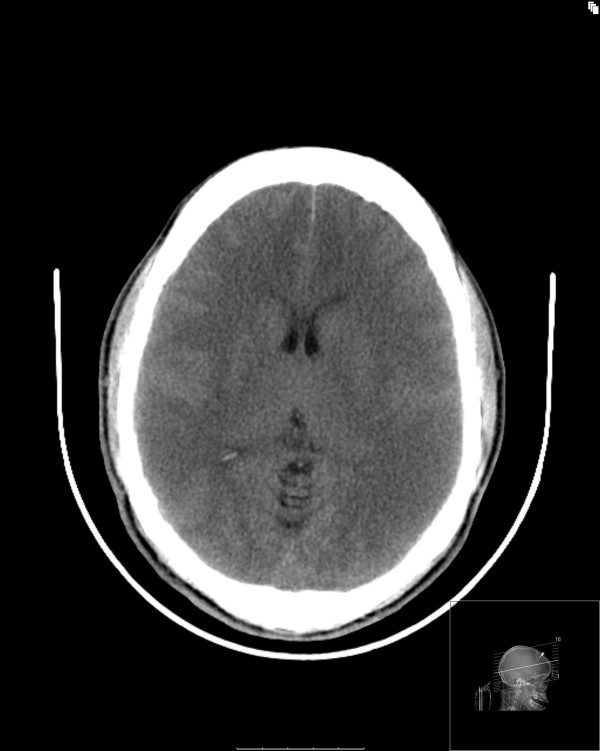
**Computed tomography image showing significant edema predominantly in the supratentorial region**.

**Figure 2:**
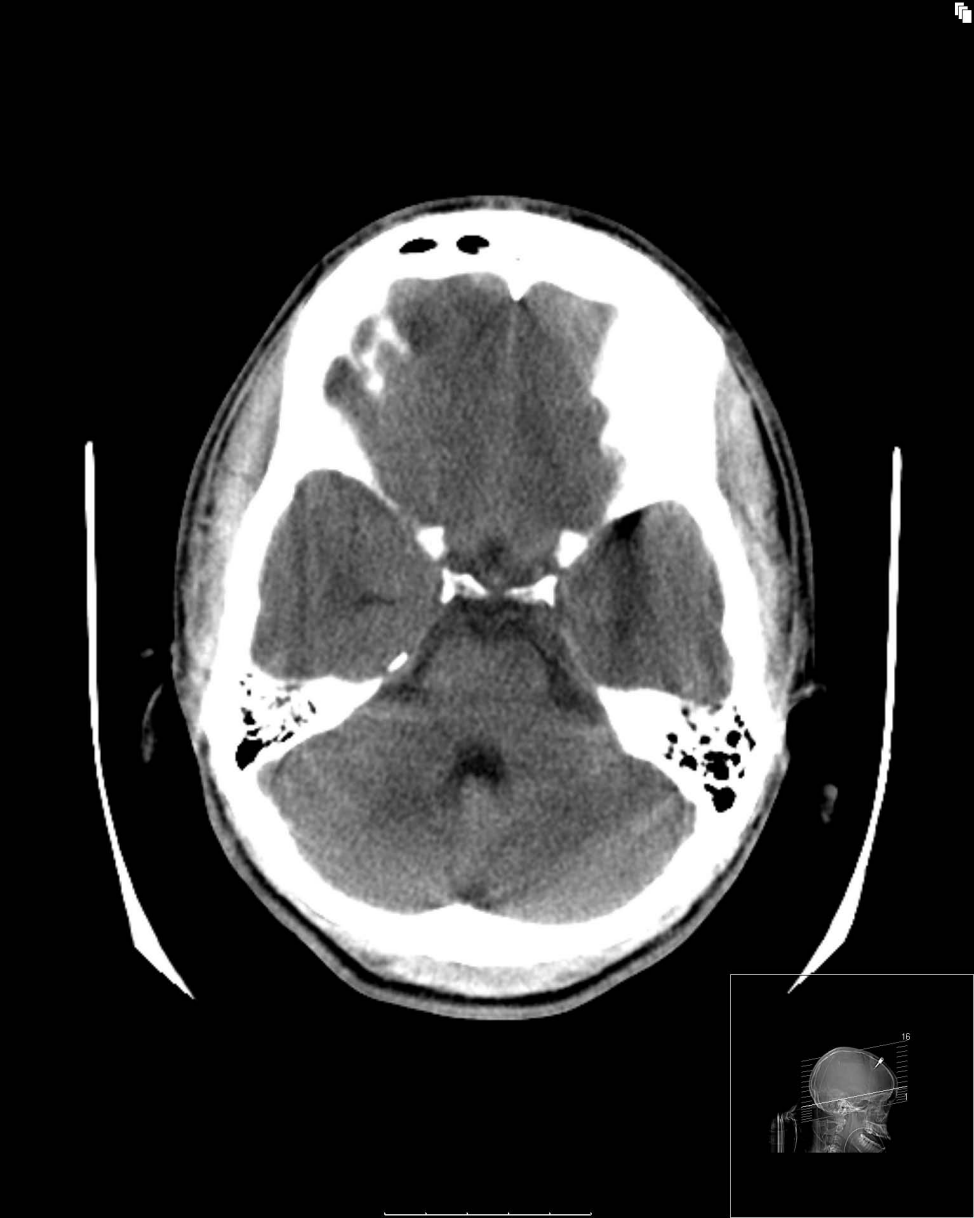
**Computed tomography image showing relative sparing of the infratentorial compartment**.

Approximately 24 hours after the onset of neurogenic diabetes insipidus, the patient's pupils became progressively more dilated and unreactive to light. His ICP, however, remained stable at 15 mm Hg. After a bolus of mannitol (0.5 g/kg), the patient was transferred to the radiology suite. A CT brain scan revealed a relatively tight supratentorial compartment with evidence of edema but with a minimal change in his infratentorial compartment and widely patent prepontine ambient cisterns. No acute bleed was identified (Figures 1 and 2).

A neurology consultation was sought because the patient now had neurogenic diabetes insipidus, fixed dilated pupils and supratentorial cerebral edema with maximal therapy, normal ICP, and no surgically treatable intracranial pathology on a background of subacute fulminant liver failure. The neurology opinion was that the patient had most likely experienced an irreversible transtentorial brain herniation despite an apparently normal ICP; thus, the likelihood of favorable neurologic recovery was minimal. A joint decision with the transplant team was then made to remove the patient from the transplant list. The patient died approximately 24 hours later.

Postmortem examination revealed "mild" cerebral edema with the presence of Alzheimer-type II astrocytes, suggesting hepatic encephalopathy. Histologic examination of the liver confirmed acute massive confluent hepatic necrosis with no evidence of chronic liver disease, possibly suggestive of an autoimmune etiology.

## Discussion

Hepatic encephalopathy in patients with fulminant liver failure is characterized by changes in cerebral blood flow, cerebral edema and intracranial hypertension. Cerebral blood flow may increase in patients with fulminant hepatic failure because of gradual cerebral arteriolar vasodilatation and may lead to cerebral hyperemia and jugular venous oxygen saturations above 75% [3]. The prognosis for patients with such cerebral "luxury perfusion" and cerebral edema is very poor [4].

Cerebral edema in patients with fulminant liver failure is a characteristically cytotoxic edema resulting primarily from astrocyte swelling. This was demonstrated in our patient during the postmortem examination [5]. Increased ICP associated with cerebral edema is a common complication of fulminant liver failure. Clinical signs of cerebral edema and intracranial hypertension in patients with metabolic encephalopathy are neither specific nor sensitive. Features commonly seen are pupillary abnormalities, exaggerated reflexes and hemodynamic instability. A head CT scan frequently demonstrates cerebral edema in acute liver failure patients with advanced-stage hepatic encephalopathy [6] but is insensitive to intracranial hypertension [7]. In addition, clinical signs of intracranial hypertension, such as decerebrate posturing and sluggish pupillary responses, have been seen at ICPs of only 15 to 16 mm Hg in patients with fulminant hepatic failure and cerebral edema [7].

Neurogenic diabetes insipidus is an uncommon condition. It is usually caused by lesions in the pituitary or supraoptic gland and paraventricular nuclei. It has also been identified in a variety of conditions. Neurogenic diabetes insipidus is defined as a decreased secretion of arginine vasopressin (AVP) leading to a high urine output of greater than 30 mL/kg per 24 hours [4]. For polyuria to become clinically apparent, 80% of AVP-secreting neurons must be destroyed. The pathophysiology of posttraumatic neurogenic diabetes insipidus may involve increased ICP and cerebral edema around the hypothalamus or posterior pituitary gland, which explains the occurrence of diabetes insipidus in our patient [1].

## Conclusions

To the best of our knowledge, there are no reports in the literature of neurogenic diabetes insipidus associated with cerebral edema from fulminant liver failure. Our patient had evidence of metabolic encephalopathy, cerebral hyperemia and cerebral edema. The cerebral edema had a relatively supratentorial distribution. The anatomical proximity of edema to the neurohypophysis most likely resulted in neurogenic diabetes insipidus.

## Abbreviations

ALT: alanine transferase; ALP: alkaline phosphatase; AVP: arginine vasopressin; CT: computed tomography; FFP: fresh-frozen plasma; ICP: intracranial pressure; ICU: intensive care unit; INR: International Normalized Ratio; IV: intravenous; PCSIMV: pressure-controlled synchronized intermittent mandatory ventilation;

## Consent

Written informed consent could not be obtained despite all reasonable attempts as next of kin could not be contacted. Every effort has been made to protect the identity of the patient and there is no reason to think that the patient or their family would object to this publication.

## Competing interests

The authors declare that they have no competing interests.

## Authors' contributions

MSH and AKP analyzed and interpreted patient data regarding the ICU management. AKB, SS, JO and BA were major contributors in writing the manuscript. All authors read and approved the final manuscript.
